# Epigenetic Modifications in Generalized Autoimmune Epithelitis: Sjögren’s Syndrome and Primary Biliary Cholangitis

**DOI:** 10.3390/epigenomes3030015

**Published:** 2019-08-08

**Authors:** Pinelopi Arvaniti, Kalliopi Zachou, Aggeliki Lyberopoulou, Nikolaos K. Gatselis, Wesley H. Brooks, George N. Dalekos, Yves Renaudineau

**Affiliations:** 1 Department of Medicine and Research Laboratory of Internal Medicine, University Hospital of Larissa, 41222 Larissa, Greece; 2 Institute of Internal Medicine and Hepatology, 41222 Larissa, Greece; 3 Laboratory of Immunology and Immunotherapy, Brest University Medical School Hospital, 29200 Brest, France; 4 Department of Chemistry, University of South Florida, Tampa, FL 33620, USA; 5 UMR1227, Lymphocytes B et Autoimmunité, Université de Brest, INSERM, CHU de Brest, 29200 Brest, France

**Keywords:** Sjögren Syndrome, sicca complex, primary biliary cholangitis, epithelitis, epigenetics, DNA methylation

## Abstract

Sjögren’s syndrome (SjS) and primary biliary cholangitis (PBC) can be classified as a model of generalized autoimmune epithelitis based on their frequent coexistence in clinical practice and the highly specific immune mediated injury of target epithelial cells. Both of these autoimmune diseases are characterized by female predominance, highly specific circulating autoantibodies, and immune-mediated destruction of the salivary and lachrymal glands and the biliary epithelial cells, respectively. Although the genetic predisposition has been well described for both diseases, genetic studies have failed to completely elucidate their pathogenesis. The recent integration of epigenetic data, analyzing the different cellular partners, opens new perspectives and allows for better understanding of these complex and still incurable diseases. Epigenetic studies on SjS have elucidated the role of DNA methylation alterations in disease pathogenesis, while epigenetic changes that influence expression of genes on the X chromosome have been implicated in the geo-variability and occurrence of PBC. The aim of this review is to describe the advances in epigenetics in the field of autoimmune epithelitis as well as to highlight how epigenetic changes could contribute to better understanding of disease pathogenesis and progression. These advances could yield insights on novel therapeutic interventions.

## 1. Introduction

Autoimmune diseases still present a great challenge for researchers especially in respect to their etiopathogenesis. Sjögren’s syndrome (SjS) and primary biliary cholangitis (PBC) represent two major models of autoimmunity both characterized by chronic immune-mediated epithelitis. SjS and PBC frequently coexist and share similar pathogenic mechanisms along with similar epidemiological features [1,2,3]. SjS is characterized by lymphocytic infiltration of lachrymal and salivary glands leading to progressive loss of secretory functions and the development of xerophthalmia and xerostomia [1]. PBC is an autoimmune cholestatic liver disease characterized by progressive inflammatory destruction of the small intrahepatic bile ducts with portal inflammation and progressive fibrosis, leading to cirrhosis and subsequent liver failure [2]. Both SjS and PBC affect mainly middle-aged women, similarly to many other autoimmune diseases, with a female to male ratio of 9–10:1. The occurrence of SjS in PBC patients is ~40%, representing a striking example of what is called autoimmune clustering [4,5,6,7,8,9,10,11]. On the other hand, approximately 20% of patients with SjS have abnormalities of liver functions [3,12,13].

Regarding autoimmune serology, both diseases are characterized by highly specific circulating autoantibodies. Indeed, antinuclear antibodies (ANA) are frequently detected in both diseases, with higher prevalence in SjS [3,12,13,14]. In this context, anti-sicca syndrome type A (anti-Ro/SSA) and anti-sicca syndrome type B (anti-La/SSB) antibodies are the characteristic autoantibodies commonly detected in patients with SjS [15]. PBC patients are characterized by the detection of serum antimitochondrial antibodies (AMA), in more than 90–95% of patients. In addition, PBC-specific ANA, such as multiple nuclear dots (MND) or rim-like membrane (RLM) pattern, are observed in ~30% of patients [5,6,9,10,16]. The main antigenic nuclear targets in PBC are directed against sp100 for the MND pattern and against gp210 for the RLM pattern. Both the MND and RLM patterns have been associated with an advanced and more severe disease course [5,6,9,10,16,17,18,19,20,21,22].

Histologically, both diseases are characterized by infiltration of B and T cells affecting exocrine glands and intrahepatic bile ducts, respectively. In SjS the infiltration of activated CD4(+), CD8(+) T, and (CD19+) B lymphocytes mainly affects the lachrymal and salivary glands leading to a progressive loss of their secretory functions and the development of the “sicca complex”, consisting of xerophthalmia and xerostomia [12,13,23,24]. In PBC, the histological hallmark is the presence of non-suppurative destructive cholangitis of the small intrahepatic bile ducts, which, at the early stages, shares numerous similarities with SjS, but progressively leads to loss of the small intrahepatic ducts [4,8,9]. The disease starts with the development of a cholestasis that may lead to liver cirrhosis with portal hypertension and end-stage liver disease [2,4,8,9,25,26]. Fatigue and pruritus are frequently observed as well as a “sicca complex” that is strongly associated with PBC [2,4,7,9,10,27,28,29,30,31].

Genetic predisposition has been well described in both diseases as attested by correlations with specific HLA and non-HLA risk loci. In SjS, HLA-DRB1*0301-DRB3*0101-DQA1*0501-DQB1*0201 was found to be associated with the development of SjS in Caucasians [32], whereas the DRB1*08032/DQA1*0103/DQB1*0601 and DRB1*08032 alleles have been reported in the Japanese population [33]. Other loci involved in a wide variety of innate and adaptive immune processes have also been shown to be substantially associated with SjS. Examples are genes encoding molecules involved in type I and type II Interferon (IFN) signaling and target processes (such as *IRF5*, *IL12A*, and *STAT4*), NF-κB signaling (for example, *TNIP1* and *TNFAIP3*), lymphocyte trafficking (such as *CXCR5*), and activation and differentiation of antibody-producing cells (such as *BLK*) [34]. Regarding PBC, HLA-DRB1*08 allele was associated with PBC development, while DRB1*11 and DRB1*13 are considered as protective alleles. In addition, genome-wide association studies (GWAS) revealed more than 40 non-HLA alleles contributing to PBC susceptibility [2,3,35]. Environmental factors such as chemicals, xenobiotics, and infectious agents have been considered to be involved in the pathogenesis of both SjS and PBC. Environmental exposures act as triggering factors, initiating an autoimmune attack in genetically susceptible hosts which might be perpetuated as observed in mechanisms involving molecular mimicry. However, genetic and environmental factors failed to firmly explain discrepancies in disease occurrence in different populations suggesting that epigenetic modifications play a significant role in disease pathogenesis [36,37,38,39,40,41].

**Figure 1:**
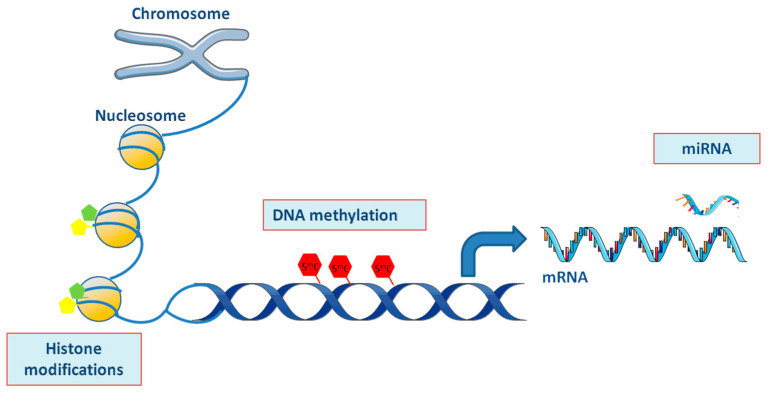
Epigenetic mechanisms include DNA methylation, histone modifications, and miRNAs. DNA methylation results from a balance between unmethylated cytosines, methylated cytosines (5^m^C), and hydroxymethylatedcytosines (5^hm^C) in CpG dinucleotides, catalyzed by DNMTs (C→5^m^C) and TETs (5^m^C→5^hm^C→C). Methylation leads to structural changes of chromatin and is associated with gene silencing. The basic unit of chromatin is the nucleosome, a nucleoprotein complex that consists of DNA wrapped around an octameric core of histones. N-terminal tails of core histones are post-translationally modified mostly by acetylation, methylation, phosphorylation, and ubiquitination (green and yellow pentagons). Histone modifications can alter DNA accessibility, facilitate RNA polymerase activity and can increase or decrease gene transcriptional activity. MiRNAs bind to mRNA and cause RNA degradation, thereby limiting the production of gene products and leading to gene silencing. Abbreviations: miRNA: micro-RNA; mRNA: messenger RNA.

Epigenetics include any heritable and functional changes in gene activity without affecting nucleotide sequences. These changes are reversible, cell type-specific, and are impacted by age, sex, and environmental factors. Three main epigenetic mechanisms coexist in a normally regulated genome: DNA methylation, histone modifications, and micro-RNAs, as shown in Figure 1 [42,43]. Among them, DNA methylation represents the most extensively studied epigenetic process in eukaryotic cells. Epigenetic control by DNA methylation results from a balance between methylation of carbon 5 of cytosine (5^m^C) in DNA CpG dinucleotides catalyzed by the DNA methyl transferases (DNMT1, 3A and 3B) and demethylation (5^hm^C) initiated by the oxidation activity of the Ten Eleven Translocation (TET1, 2 and 3) methylcytosinedioxygenase [42,44,45].

Epigenetic studies in SjS revealed different methylation patterns in salivary gland epithelial cells (SGECs) and inflammatory cells [46,47], while methylation alterations were correlated with the number of infiltrating lymphocytes and also with the expression of methylation/demethylation enzymes [48]. In addition, epigenome-wide association studies (EWAS) highlighted the hypomethylation of IFN regulated genes both in SGECs and immune cells [49]. In the field of PBC, studies are just beginning to explore the role of epigenetics in disease pathogenesis [50,51,52,53,54,55,56]. The most intriguing data arise from the evaluation of the X chromosome, which revealed hypomethylation of several gene promoters in women.

Thus, the aim of this review is to summarize recent findings regarding epigenetic alterations in SjS and PBC that could potentially open new perspectives of their pathogenesis and could yield new insights to the development of novel therapeutic strategies.

## 2. Epigenetic Modifications in SjS and PBC

### 2.1. DNA Methylation/Hydroxymethylation

#### Description

DNA methylation is the most studied epigenetic factor in eukaryotic cells, and involves the addition of a methyl group at position 5 of the cytosine pyrimidine ring to form 5^m^C within CpG base pairs. Such addition occurs predominantly in the CpG islands that have been conserved throughout evolution within the regulatory regions in up to 70% of human genes [42,43]. The methylation status of CpG islands leads to structural changes of chromatin, which can affect transcription. For example, when methylated, the promoter recruits methyl-CpG-binding proteins, such as methyl-CpG-binding protein 2 (MeCP2) and methyl-CpG-binding domain protein 2 (MBD2), which then recruit chromatin inactivation complexes containing histone deacetylases (HDACs) and histone methyltransferases (HMTs). The methylation process is catalytically mediated by DNMTs (DNMT1, DNMT3A, and DNMT3B), and uses S-adenosylmethionine (SAM) as the methyl donor compound [42,43,57].

An active DNA demethylation process was recently described involving the TET enzyme family (TET1,2,3), which oxidizes 5^m^C into 5-hydroxymethylcytosine (5^hm^C) and, subsequently, in a less efficient fashion, into 5-formylcytosine (5-fCyt) and 5-carboxylcytosine (5-CaCyt) with the use of α-ketoglutarate (α-KG), molecular oxygen, and iron as cofactors [44]. DNA methylation and active DNA demethylation processes are not restricted to DNMTs and TETs. Other models have demonstrated that 5^hm^C can be enzymatically deaminated by activation-induced deaminase (AID)/apolipoprotein B editing complex (APOBEC) to 5-hydroxymethyluracil (5^hm^U) which can then be processed by various DNA glycosylases, including methyl-CpG-binding domain protein 2 (MBD4), generating an unmodified cytosine [44,45,48].

### 2.2. DNA Methylation/Hydroxymethylation in SjS and PBC

DNA methylation/hydroxymethylation represents one of the main epigenetic mechanisms that have been studied in the field of SjS. Studies on minor salivary gland (MSG) biopsies, and long-term cultured SGECs from patients with SjS have shown reduced levels of global DNA methylation (5mC) on epithelial cells whereas no differences were noticed in B and T lymphocytes [45,46,47,48,57,58,59,60]. Moreover, in MSG from SjS patients, the DNA methylation level in epithelial cells is inversely correlated with the number of infiltrating lymphocytes. It is further suspected that B lymphocytes control DNA methylation in epithelial cells as this defect is reversed after B cell depletion treatment [59,61]. At the molecular level, the DNA methylation deficit was correlated with lower DNMT1 expression through alterations in the Erk/DNMT1 pathway and also with increased expression of the demethylating partner Gadd45α [58,59]. More recently, Lagos et al. reported a decrease in DNA methylation (5mCyt) in MSG epithelial cells from patients with SjS, and this reduction was associated with a decrease of MeCP2, a repressive factor [62]. Gonzalez’s group has further reported, in MSG, a lower level of 5^m^C and DNMTs within leucocytes than in epithelial cells. In addition, the active DNA demethylation process is also affected as epithelial cells have an increase in 5^hm^C level, an effect which was attributed to the overexpression of TET2. In a human salivary gland epithelial cell line, TETs are controlled through the activation of a Jak/STAT3 pathway mediated by proinflammatory cytokines (type I and type II interferon [IFN]) and reactive oxygen species [63].

A defective methylation of the long interspersed nuclear element 1 (LINE-1) promoter was identified in MSG from SjS patients. LINE-1 elements are retroviral-like, and with endogenous DNA sequences that are able to amplify and transpose to new locations in the genome, through an RNA intermediate. LINE-1 elements are over-expressed in MSG biopsies from patients with SjS and the expression is linked to type I IFN production [64]. LINE-1 expression, in MSG, was inversely correlated with the methylation status of CpGs of its promoter suggesting that DNA methylation status controls LINE-1 expression in those tissues. In addition, amore recent study from Mavragani et al., demonstrated a reduced level of LINE-1 promoter methylation along with increased DNMT3B, DNMT1, and MeCP2 in SjS patients with low risk for developing lymphoma compared to both SjS-lymphoma patients and sicca controls [65].

In PBC, several studies have shown that differences in methylation in the X chromosome may be involved in disease pathogenesis (Table 1). In females, one of the two X chromosomes is inactivated by DNA methylation in order to have equal expression of X-linked genes between males and females. However, in some diseases, this compensatory mechanism is defective as demonstrated in CD4 (+) T cells from women with PBC that present hypomethylation of the CD40L promoter located on the X chromosome at position q26, and a higher expression of CD40L in the absence of mutations in the CD40L gene [66]. Moreover, CD40L promoter hypomethylation is correlated with IgM hypergammaglobulinemia and has been implicated in autoimmunity through three main mechanisms: (i) a decrease in clearance of autoreactive T cells, (ii) an increase in T cell priming, and (iii) upregulation of inflammatory cytokines. The same observation was reported for CXCR3, another X chromosome gene, as its promoter is demethylated in CD4(+) T lymphocytes from women with PBC [51]. CXCR3 is integral in Th1 cell differentiation as it regulates interactions within lymph nodes between antigen-specific CD4(+) T cells and dendritic cells presenting the same antigen [50,51]. Significant differences in DNA methylation have also been found in other gene promoters located in the X chromosome of CD4(+), CD8(+), and CD14(+) cells [51]. In particular, FUN14 Domain Containing 2 (FUNDC2) was hypermethylated in CD8(+) T cells, which resulted in a significant decrease in gene expression. The X-linked interleukin-1 receptor accessory protein-like 2 (*IL1RAPL2*) and the Ubiquitin-conjugating enzyme E2 A (*UBE2A)* were also hypermethylated in CD4(+) T cells and CD14(+) cells, respectively, but no difference in gene expression was detected [51,67].

### 2.3. Genome-Wide Analysis of DNA Methylation in SjS and PBC

EWAS enable the analysis of DNA methylation of site specific CpGs. All studies conducted so far in SjS used the 450K array from Illumina. The 450K array technology has limitations. One limitation is that the 450K array does not allow quantification of global DNA methylation. Another limitation is that both DNA methylation and hydroxymethylation are analyzed together. To the best of our knowledge, two groups have studied the methylation status of different CpG sites in MSG. In the first study, conducted by Cole et al., bioinformatic analysis highlighted an extended region of hypomethylation surrounding the promoters of Proteasome subunit beta type-8 (*PSMB8*) and Transporter associated with Antigen Processing 1 (*TAP1*), consistent with an increased frequency of antigen-presenting cells found in MSG from SjS patients compared to a control group [60]. Results from this study also highlighted the fact that hypomethylation of specific CpGs affects mostly genes from the IFN pathway, confirming previous findings that the IFN pathway plays an important role in SjS pathogenesis [70]. In the second study conducted by Imgenberg-Kreuz et al., the methylation profiles of multiple tissues (whole blood, CD19(+) B cells, and MSG biopsies) were analyzed in SjS together with gene expression in CD19(+) B lymphocytes [71]. According to this study, 45 differentially methylated CpGs (DMCs) corresponding to 19 unique genes were identified in MSG from SjS patients. The most significant DMCs showed hypomethylation in 2′-5′-oligoadenylate synthetase 2(*OAS2*), which encodes a member of the IFN-inducible 2′-5′ synthetase family, involved in the innate immune response to viral infections.

However, the limitation of using MSG biopsies was the heterogeneity of cell types, as MSG tissues consist of both epithelial and inflammatory cells with different methylation patterns reported between different cell populations. To overcome this limitation, Charras et al. conducted a whole genome methylation analysis in long-term cultured SGECs which revealed 4662 DMCs between patients and controls, corresponding to 2560 unique and annotated genes. The results of this study confirmed that epigenetic changes affect mostly IFN regulated genes, but also highlighted the enrichment of the calcium pathway (implicated in saliva production) and the Wnt pathway (implicated in SGECs differentiation and survival) for hypomethylated and hypermethylated DMCs, respectively [49].

A number of genes previously shown to be differentially methylated in SGECs were also found in T and even more in B lymphocytes suggesting that similar pathways may be affected in diverse cellular subsets [49]. In particular, methylation alterations in lymphocytes affect mostly, as in SGECs, genes regulated by IFN signaling such as signal transducer and activator of transcription 1 (*STAT1*), interferon-induced transmembrane protein 1 (*IFITMI)* and interferon-induced protein 44 like (*IFI44L*) [49,70,72,73,74]. Moreover, the hypomethylation of IFN regulated genes was associated with an increase in gene expression in CD19(+) B lymphocytes [67], and a more prominent difference was associated with disease activity and anti-SSA/B positivity [72]. Apart from IFN regulated genes, DNA methylation modifications also affect genes involved in lymphocytic activation and immune response, such as the genes of lymphotoxin A and forkhead box protein P3(*FOXP3*) as well as the tumor necrosis factor ligand superfamily member 7 (*TNFSF7*) gene promoter in T cells. Genes related to B cell signaling in B cells and genes correlated with a predisposition to lymphoma development, such as runt-related transcription factor 1 (*RUNX1*), are also observed to be hypomethylated [72,74,75,76].

Finally, integration of genome-wide association studies (GWAS) and epigenome-wide associated studies (EWAS) revealed a significant link between SjS-associated genetic risk factors and DMCs in SGECs as well as in B and T lymphocytes [49,67,72,73]. Methylation quantitative trait loci (meQTL) of genetic variants within HLA and non-HLA regions, including IFN regulated genes, and DNA methylation were identified (e.g., IRF5, CXCR5, and HLA-DQB1), indicating that SjS risk factors potentially affect DNA methylation-sensitive pathways at target CpG sites [67].

As far as PBC is concerned, Selmi et al. [69] performed EWAS in three pairs of monozygotic female twins and eight pairs of sisters discordant for PBC. Although the number of patients included in the study was limited, the authors found 60 and 14 gene regions with different methylation between the discordant twin sets and between PBC cases and control sisters, respectively. Interestingly, 51 of 60 genes were mapped to the X chromosome, which is in agreement with the female predominance of the disease [50,66,69] and a defective X chromosome compensatory mechanism (see before). Methylation modifications were linked to altered gene expression levels and revealed that five genes (*CXCR5*, *HLA-B*, *IFI44L*, *IFITI*, and *SMARCA1*) were downregulated and 1 gene (*IL6*) was upregulated in PBC. In particular, these genes cause the downregulation of Th2-cytokines such as *IFIT1*, an IFN type I signature represented by *IFI44L*. However, methylation did not completely correlate with the expression levels of most genes, indicating that other mechanisms, such as allele-specific methylation may be involved in gene transcription [73]. In addition, extensive methylation profiling of the X chromosome by bisulfite sequencing revealed a total of 20, 15, and nine distinct gene promoters with a significant difference in the methylation profile in CD4(+), CD8(+), and CD14(+) cells in PBC patients, respectively, further implicating epigenetic alterations in disease pathogenesis [51,66].

Results from these studies support the idea that the female predominance in PBC is reflected by the number of DMCs located on the X chromosome. In SjS, Mougeot et al. proposed that the upregulation of X chromosome genes reported in SjS could be explained by a defective compensatory mechanism based on the analysis of gene expression and EWAS datasets [77]. Such an hypothesis is further reinforced by the fact that X chromosome aneuploidies (47,XXX and 47,XXY) represent a risk factor for developing SjS [78,79].

### 2.4. Histone Modifications

#### Description

The basic unit of chromatin is the nucleosome, a nucleoprotein complex that consists of DNA wrapped around an octameric core of histones (two each of histones H2A, H2B, H3, and H4). Histones are small globular proteins with flexible N-terminal tails that project from the nucleosome core. Epigenetic modification sites in the chromatin structure consist primarily of lysine, arginine and serine residues in the N-terminal tails of core histones. The residues are post-translationally modified mostly by acetylation, methylation, phosphorylation, and ubiquitination. As a consequence, the N-terminal tail modifications can increase or decrease the interactions of the histones with neighboring histones, DNA molecules, and nuclear proteins, and thereby affecting gene transcription [80]. How histone modifications affect gene activity is referred to as the histone code and their positive or negative effect depends on the specific nature and location of the amino acid residues on the histone. Generally, histone modifications tend to destabilize the genome, and they have been associated with the loss of self-tolerance in immune cells and the promotion of an autoimmune propensity [81,82].

### 2.5. Histone Modifications in SjS and PBC

To the best of our knowledge, no public datasets on histone modifications rising directly from primary cells from patients with SjS are currently available. However, an enrichment of differentially methylated sites in SjS patients overlapping with specific histone modifications was found in reference B and T cells [67,73]. In more detail, in whole blood from patients with SjS, hypomethylated CpG sites were overrepresented in enhancer regions with H3K4me1 and H3K27ac, whereas hypermethylated sites were underrepresented in the same regions, which, contrarily, were enriched with the histone mark for actively transcribed genes: H3K36me3 [83]. Moreover, simultaneous genomic–epigenomic analysis showed that susceptibility variants were specifically associated with histone marks for promoters and enhancers in reference B cells, and with enhancers in reference monocytes [75], underlying the potential pivotal role of histone modifications in SjS pathogenesis.

Histone modifications are better described in PBC. The integral role of CD40L in PBC, is supported not only by the hypomethylation of its promoter, but also by the increased H4 histone acetylation of its promoter which may also lead to increased expression of CD40L [66,67]. Additional histone modifications including increased H4 acetylation of *LIGHT*, *IL-17*, and *IFNγ* gene promoters also characterize autoreactive T cells in PBC. Importantly, *LIGHT* is critical in autoimmunity, as it is a co-stimulatory molecule vital for the control of T cell proliferation [68,84]. On the contrary, deacetylation of tumor necrosis factor (TNF)-related apoptosis-inducing ligand (*TRAIL*), *Apo2*, and *HDAC7A* promoters has been described. *TRAIL* is integral in the induction of apoptosis, but is also thought to inhibit autoimmunity through cell cycle arrest under certain conditions. Therefore, *TRAIL* is supposed to play a dual role in PBC, promoting apoptosis and the development of autoantibodies, but also the susceptibility to autoimmunity [68,70]. Finally, Farh, et al. integrated genetic and epigenetic fine mapping of histone marks to identify causal variants in autoimmune diseases and found that, in PBC, differentially methylated CpG sites are associated with histone modifications which are preferentially located at enhancers and promoters in B cells [83].

### 2.6. Micro-RNAs (miRNAs)

#### Description

The miRNAs are small noncoding, single-stranded RNAs of 19 to 22 nucleotides in length, which regulate gene expression at the post-transcriptional level. miRNAs are generated in the nucleus as primary mRNA transcripts by RNA polymerase II and afterwards they are transported to the cytoplasm for further processing into mature miRNAs [85]. Most miRNAs bind to the 3′ untranslated region (UTR) of the targeted mRNAs which leads to either mRNA degradation or repression. Dysregulation of miRNA expression is found to be associated with the onset and progression of autoimmune diseases including SjS and PBC [45,85,86].

### 2.7. miRNAs in SjS and PBC

Several studies have shown altered miRNA expression in SjS patients compared to controls (Table 2) [87,88,89,90,91,92,93,94]. The predictive analysis of biologic pathways under miRNA control, suggests regulation of the neurologic pathways controlling salivation, as well as the lack of transcriptional regulation of the two main autoantigens SSA/Ro and SSB/La by the miRNAlet-7b, which is repressed in SjS [95,96,97,98,99,100]. Furthermore, studies in peripheral blood mononuclear cells (PBMCs) have shown that miR146a and miR155 are upregulated in response to the adaptive immune response in SjS both in mice and humans. Interestingly, miR146a is increased prior to disease onset and during full-blown SjS in the salivary glands, suggesting that miR146a may be involved in early disease pathogenesis. miR146a plays a critical role in increasing phagocytic activity and repressing inflammatory cytokine production in human monocytic Th1 cells through a Toll-like receptor (TLR)/IFN pathway [91,100]. Through exosomes, miRNAs can be transferred from B cells to SGEC as reported with EBV-miR-BART13-3p, which targets the calcium sensor *STIM1*, a primary regulator of the calcium signaling pathway [99,101,102].

Regarding PBC, so far, more than 200 miRNAs have been identified in PBMCs and liver specimens from patients (Table 3). The first study was conducted in 2009, and revealed the downregulation of miR-122a and miR-26a and the increased expression of miR-328 and miR-299-5p [103]. Many studies were performed thereafter [86,104,105,106,107,108,109,110,111,112,113,114], one of which proposed an miRNA panel consisting of hsa-miR-122-5p, hsa-miR-141-3p, and hsa-miR-26b-5p for disease diagnosis [106]. However, only the study by Banales et al. [110] revealed for the first time a functional role of miRNAs in the immunopathogenesis of PBC by linking miR-506 with the hypothesis of defective “biliary bicarbonate umbrella”, which is believed to play a central role in PBC pathogenesis [115]. The electroneutral Na^+^-independent Cl^−^/ HCO3^−^ anion exchanger 2 (AE2) is expressed in human cholangiocytes, and in particular in their apical domain being the main biliary HCO3^−^ extruder [116,117,118]. The net result is the generation of a luminal alkaline “umbrella” avoiding the hydrophobic bile salt monomers that are toxic for the cholangiocytes and induce cytotoxicity [119,120,121]. In PBC, decreased AE2 expression and activity has been found in PBC liver biopsies and PBMCs compared to healthy controls. This condition may promote firstly, an impaired biliary bicarbonate secretion with consequent cholestasis and cholangiocyte distress, and secondly, the breakdown of immune tolerance leading to an attack against the susceptible cholangiocytes giving rise to extended biliary damage and ductopenia [86,122,123,124].

In this context, Banales et al. [110] showed that miR-506 could be a potential contributor of PBC pathogenesis as its overexpression impairs biliary bicarbonate secretion through direct targeting of not only AE2, but also type III inositol 1,4,5-trisphosphate receptor (InsR3P3) that functions as a calcium (Ca^2+^) release channel at the endoplasmic reticulum membrane of cholangiocytes, contributing to the bicarbonate secretion via AE2. On the other hand, molecular targeting of miR-506 with complementary antisense oligonucleotides resulted in the restoration of AE2 activity in PBC cholangiocytes in vitro [123]. The exact molecular mechanisms that promote miR-506 overexpression in PBC remain to be elucidated. The fact that miR-506 is located on the X-chromosome and that PBC mainly affects females further emphasizes the potential role of X chromosomes in PBC. From the clinical point of view, as anti-miR treatments have been reported efficient and well-tolerated in other liver disease patients, such as patients with hepatitis C [125], their efficacy in PBC should be evaluated. Of note, miR-506 upregulation was confirmed in PBC livers from an independent cohort of patients compared to healthy controls and patients with primary sclerosing cholangitis [110,112].

## 3. Overview and Future Perspectives

The integration of epigenetic mechanisms in the pathogenesis of SjS and PBC opens new insights towards a better understanding of the etiology of these diseases and their progression, and also towards the potential establishment of novel therapeutic interventions. The general findings of epigenetic studies in SjS and PBC is DNA hypomethylation of promoter regions at IFN-regulated genes in epithelial cells from MSG and B cells and in genes located on the X chromosome in T cells, respectively. Focusing on these findings, several therapeutic targets can be identified.

In autoimmune diseases, regulatory genes are mainly hypomethylated and histone marks acetylated; therefore, transcriptional activity is increased along with circulating miRNA levels. As a consequence, epigenetic treatments should be able to reduce transcriptional activity of targeted genes. DNMTs, TETs, histone deacetylases, and miRNAs represent potential targets for epigenetics-related treatments that have already started to evolve in the fields of chronic infections and malignant diseases [126,127,128,129,130]. However, in the field of autoimmunity, the inhibition of MBDs and histone deacetylase activity has controversial effects, while modulation of miRNAs is just beginning as a new therapeutic tool [131,132]. Regarding epigenetic modifiers, such as DNMT inhibitors and miRNA antagonists, they are in clinical use or in clinical trials in cancer [129], but no clinical studies affecting epigenetic modulation are currently under way for SjS or PBC. Overall, the therapeutic epigenetic interventions still lack precision, disease specificity and proven safety. A major concern is that the manipulation of these complex mechanisms may have unsuspected and unwanted consequences. Furthermore, the manipulated epigenetic change may become an inheritable trait that involves subsequent generations. This means that further investigations are warranted so that new and safe epigenetic-based treatment strategies can be established.

For this purpose, the choice of relevant cell and tissue types is crucial. Whole blood, although an easily accessible tissue, is composed of many different cell types, and results may be affected by the underlying heterogeneity in cell type composition between patients and controls. In addition, biopsies from affected tissues are also composed of mixed cell types, and reference epigenetic data sets are not available for these tissues. In order to fully determine the contribution of epigenetic changes in different cell subtypes the analysis of pure cell populations is needed. The study of long-term cultured cells from affected tissues is a potential approach; however, such approaches are complicated by the difficulty to obtain these tissues, which typically leads to limited, smaller sample sizes. However, as costs for DNA sequencing gare coming down, whole-genome bisulfate sequencing studies and studies of histone modifications in multiple tissues from large numbers of SjS patients will soon be feasible. In an attempt to fully clarify the role of DNA methylation in SjS and PBC, further epigenetic studies are warranted, which will potentially identify new biomarkers for diagnosis, patients stratification according to prognosis and novel perspectives for therapeutic interventions [86,133].

## 4. Conclusions

In conclusion, epigenetic alterations in autoimmune epithelitis in SjS and PBC provide insights for better understanding of the diseases’ pathogenesis. However, the epigenetic features must be associated with disease occurrence and clinical manifestations while their functional consequences need to be fully investigated so that new, effective, and safe treatment manipulations can be established.

## Figures and Tables

**Table 1:** Studies on DNA methylation and histone modifications in PBC.

Study	Method	Sample	Findings
Huet al, 2011 [68]	Chromatin Immunoprecipitation (ChIP)	60 PBC patients (55 women, 5 men)	Increased H4 histone acetylation of promoters for CD40L, LIGHT, IL-17, IFNγ and deacetylation of H4 histone of TRAIL, Apo2, HDAC7A promoter in T cells.
Lleo et al., 2012 [66]	Bisulfite sequencing	20 PBC women, 20 healthy women, 9 women with psoriasis, 9 women with DM1	Hypomethylationof CD40L gene promoter
Selmi et al., 2014 [69]	DNA immunoprecipitation (MeDIP)	3 pairs of monozygotic female twins, 8 pairs of sisters discordant for PBC	Differentmethylation • twins—60 gene regions • sisters—14 gene regions Downregulation of: CXCR5, HLA-B, IFI44L, IFIT1, and SMARCA1, Upregulation of IL-6
Lleo et al., 2015 [51]	Bisulfite sequencing	30 women with PBC30 healthy women	20, 15, and 9 distinct gene promoters with a significant difference in methylation profile in CD4(+), CD8(+), and CD14(+) T cells respectively

Abbreviations: PBC: primary biliary cholangitis; DM1: diabetes mellitus type 1; LIGHT: tumor necrosis factor superfamily member 14; IL-17: interleukin 17; IFNγ: interferon γ; TRAIL: TNF-related apoptosis-inducing ligand; Apo2: apoptosis 2; HDAC7: histone deacetylase 7; CXCR5: C-X-C motif chemokine receptor 5; HLA-B: human leucocyte antigen B; IFI44L: interferon-induced protein 44 like; IFIT1: interferon-induced protein with tetratricopeptide repeats 1; SMARCA1: SWI/SNF-related, matrix-associated, actin-dependent regulator of chromatin, subfamily A, member 1; IL-6: interleukin 6.

**Table 2:** Different miRNAs expression in SjS.

Authors	miRNAs	Tissue	Design	Findings
Michael et al., 2009 [95]	hsa-miR-203 hsa-miR-768-3p hsa-miR-574-3p	saliva	SjS vs. controls	exosomal microRNAs as disease biomarkers
Pauley et al., 2011 [103]	miR-146a	PBMC	SjS vs. controls	Overexpression of miR-146 and miR-155
Alevizos et al., 2011 [88]	miR-768-3p miR-574-3p	MSG	SjS vs. controls	miR-768-3p and miR-574 inversely correlated with focus score
Kapsogeorgou et al., 2011 [89]	miR-16 miR-200b, Let-7b miR-223 miR-181a	PBMCs, SG, SGEC	SjS vs. sicca controls and seronegative SjS patients	downregulation of let-7b in SGECs of antibody-positive patients
Tandon et al., 2012 [90]	miR-4524 miR-5100	MSG	SjS vs. controls	differential expression of miR-5100
Zilahi et al., 2012 [91]	miR-146a miR-146b	PBMCs	SjS vs. controls	Overexpression of miR-146a/b
Shi et al., 2014 [96]	miR-146a miR-155	PBMCs	SjS vs. controls	Overexpression of miR-146a, downregulation of miR-155
Peng et al., 2014 [97]	miR-181a	PBMCs	SjS vs. controls	Overexpression of miR-181a
Gourzi et al., 2015 [98]	miR16, miR200b-3p, miR223, miR483-5p	MSG, SGEC, PBMC	SjS vs. sicca controls	dysregulation of miR-16, miR-200b-3p, miR-223and miR-483-5p
Gallo et al., 2016 [99]	miR-BART13-3p	MSG	SjS vs. controls	elevation of miR-BART13-3p affects calcium pathway
Yang et al., 2016 [100]	miR-1207-5p, miR-4695-3p	MSG	SjS vs. controls	downregulation of miR-1207-5p and miR-4695-3p expression
Chen et al., 2017 [92]	miR-150-5p	PBMC	SjS vs. controls	reduced miR-150-5p expression
Wang et al., 2017 [93]	miR-181a miR-16	MSG	SjS vs. controls	decreased miR-181a/miR-16 expression associated with focus score
Wang-Renault et al., 2018 [94]	miR-30b-5p	CD19(+) B, CD4(+) T cells	SjS vs. controls	miR-30b-5p inversely correlates with BAFF expression in B cells

Abbreviations: SjS: Sjögren’s syndrome; PBMC: peripheral blood mononuclear cells; miRNAs: micrο-RNAs; MSG: minor salivary gland; SGEC: salivary gland epithelial cells.

**Table 3:** Differentially expressed miRNAs in PBC.

Authors	miRNAs	Tissue	Design	Findings
Padgett et al., 2009 [104]	miR-122a, miR-26a, miR-328, miR-299-5p	Liver	PBC vs. controls	Downregulation of miR-122a and miR-26a and the increased expression of miR-328 and miR-299-5p
Banales et al., 2012 [110]	miR-506	Liver	PBC vs. controls	miR-506 overexpression impairs biliary bicarbonate secretion
Qin et al., 2013 [105]	miR-15a-5p, miR-20a-5p, miR-140-3p, miR-106b-5p, miR-3654, miR-181a-5p	PBMCs	PBC vs. controls	altered expression of 17 miRNAs involved in cell differentiation and signal transduction
Ninomiya et al.,2013 [106]	hsa-miR-505-3p, miR-197-3p	Sera	PBC vs. controls	decreased expression of hsa-miR-505-3p and miR-197-3p
Tan et al., 2014 [109]	hsa-miR-122-5p, hsa-miR-141-3p, hsa-miR-26b-5p	Sera	PBC vs. controls	hsa-miR-122-5p, hsa-miR-141-3p, and hsa-miR-26b-5p as disease biomarkers
Sakamoto et al., 2016 [107]	miRNA-122, miRNA-378, miRNA-4311, miRNA-4714-3p	Sera	Treatment effective vs. resistant patients	miRNA profile can be a useful approach for the characterization of PBC development
Liang et al., 2016 [108]	miR-92a	Sera, PBMCs	PBC vs. controls	altered miR-92a expression is associated with Th17 cell differentiation

Abbreviations: PBC: primary biliary cholangitis; PBMCs: peripheral blood mononuclear cells; miRNas: micrο-RNAs.

## Abbreviations

SjS	Sjögren’s Syndrome
PBC	primary biliary cholangitis
ANA	antinuclear antibodies
AMA	antimitochondrial antibodies
SGECs	salivary glands epithelial cells
5^m^C	5-methylcytosine
DNMT	DNA methyltransferase
TET	ten eleven translocation methylcytosine dioxygenase
MBDs	methyl binding domains
MeCP	methyl-CpG-binding proteins
LINE-1	long interspersed nuclear element
5^hm^C	5-hydroxymethylcytosine
IFN	interferon
GWAS	genome-wide association studies
CXCR3	C-X-C motif chemokine receptor 3
EWAS	epigenome-wide association studies
DMCs	differentially methylated CpGs
HLA-B	human leucocyte antigen B
IFI44L	interferon-induced protein 44 like
IFIT1	interferon-induced protein with tetratricopeptide repeats 1
SMARCA1	SWI/SNF-related, matrix-associated, actin-dependent regulator of chromatin, subfamily A, member 1
IL-6	interleukin 6
LIGHT	tumor necrosis factor superfamily member 14
IL-17	interleukin 17
PBMCs	peripheral blood mononuclear cells
TRAIL	TNF-related apoptosis-inducing ligand
miRNAs	Micro-RNAs
UTR	untranslated region
